# Bridging the gap between objective and subjective well-being among first generation immigrants: exploring the role of religion and spirituality

**DOI:** 10.3389/fsoc.2025.1539686

**Published:** 2025-04-16

**Authors:** Samadara Batuwanthudawa, Samitha Udayanga

**Affiliations:** ^1^Department of Sociology, University of Ruhuna, Matara, Sri Lanka; ^2^Bremen International Graduate School of Social Sciences, University of Bremen, Bremen, Germany

**Keywords:** linking and relinking, objective well-being, religious affiliations, spirituality, social capital, subjective well-being

## Abstract

The role of religion and spirituality in fostering resilience among immigrants has been widely acknowledged. However, existing studies often emphasize religious affiliations established within the host country, overlooking the significance of religious practices originating from immigrants’ home countries and their unique influence on the life experiences of immigrants. The present study thus addresses this research gap by investigating how religious affiliations rooted in immigrants’ home country based traditions influence their subjective well-being in a new social context. Using a qualitative approach, we examined the experiences of Sri Lankan immigrants settled in Italy. The thematic analysis identified two key themes. The first theme highlights that, although religious practices may initially appear less significant upon immigration, their importance resurfaces during periods of uncertainty. By providing psychological stability, these practices enhance both objective and subjective dimensions of well-being. The elevated levels of happiness observed among immigrants often reflect strong affiliations with home country based religious institutions and spirituality aligned closely with home country cultural values. The second theme demonstrates that religion functions as a cultural bridge, enabling immigrants to transfer familiar traditions and practices into their new environment. Overall, the process of reconnecting with home country based religious practices and spirituality allows immigrants to navigate the host country effectively, balancing the establishment of objective well-being with the preservation of valued cultural identities, and ultimately enhancing their subjective well-being.

## 1 Introduction

Voluntary migration, in which immigrants intentionally choose to move to a country with higher living standards, is considered a viable means of achieving a better quality of life that is possibly difficult to attain in their home country (Nowok et al., 2013). The integration of these immigrants into host societies is crucial, as successful integration enhances their overall well-being. Sociocultural integration encompasses several dimensions, including civic participation, labor market integration, and engagement in cultural life (OECD/European Commission, 2023). At the same time, immigrants often develop a hybrid identity, blending characteristics from their home country with their evolving immigrant identity. The main intention of those voluntary migrants is to establish overall well-being, which consists of both objective quality of life and subjective well-being. While several studies have examined how immigrants navigate host countries and work toward well-being (Bartram, 2013; Betz and Simpson, 2013; Zafar and Ammara, 2023), little focus has been given to the influence of cultural ties with their home country on their present lives and well-being. Among these cultural ties, religious beliefs originating from home countries play a particularly significant role. Therefore, this study explores how immigrants’ adoption of religious affiliations connected to their home country may contribute to their subjective well-being in the host country.

Previous studies in this regard show that immigrants across Europe typically report higher happiness and life satisfaction compared to natives (Arpino and de Valk, 2018; Brockmann, 2021; Zafar and Ammara, 2023), and those studies have consistently demonstrated the positive association between religiosity and subjective well-being. However, research exploring how immigrants adopt and utilize their home country based religious beliefs to build resilience and achieve well-being in the host society remains limited. Even though several limitations exist, the main goal of this study is to examine how a particular immigrant community strives to navigate the host society by way of cultural means in order to achieve their desired subjective well-being. We aim to focus on Sri Lankan immigrants in Italy and examine how home country based religious beliefs are linked to their present day lives, which is likely to support achieving immigration aspirations and securing subjective well-being. Although several determinants contribute to the subjective well-being of immigrants, we particularly focus on how religious affiliations associated with their home country can foster a sense of community and support their well-being.

## 2 Literature review

Subjective well-being, broadly known as happiness, refers to how an individual experiences and evaluates quality of life (Diener et al., 1985; Paparusso, 2021; Voukelatou et al., 2021). It is a significant predictor of health, wellness, and longevity. Subjective well-being is defined as a state of life satisfaction characterized by positive and low levels of negative emotions (Diener, 2009; Durayappah, 2011). This concept is more closely related to the experiential dimension of subjective well-being rather than its evaluative aspect. Quantitative studies often focus on evaluative happiness, measuring it on a scale. In contrast, this study examines the experiential dimension of happiness, which refers to how individuals personally experience it (Diener, 2009). Such happiness is largely a cultural phenomenon, shaped by past experiences, present life circumstances, and future life prospects.

Voluntary migration is often pursued as a way toward happiness, with several contributing factors including economic stability, health, integration level, and education, among others (Hendriks, 2015; Hendriks and Bartram, 2019). There are certain cases where individuals migrate due to emergencies; however, this study does not focus on such cases. Voluntary migration, such as migration for economic purposes or family reasons, is often driven by the goal of achieving a higher quality of life that is perceived to be unattainable in their home country (Hendriks, 2015). Successful integration into the host country and securing objective well-being become the initial steps in their life trajectory. Objective well-being is more apparent and closely associated with migration aspirations, as it leads to a higher quality of life. It encompasses several dimensions, including good health, increased income, improved living standards, and a favorable socio-economic and political environment (Bartram, 2013; Voukelatou et al., 2021). One of the main drivers of migration is the aspiration to increase income and lead a satisfactory life, which may be unattainable in the country of origin (Hendriks, 2015). Therefore, the collapse of objective well-being could lead to precarity, and immigration might not yield the expected outcomes. In a way, objective well-being is at the core of immigration journeys, although happiness also guide immigrants toward objective well-being (Stranges et al., 2021). However, some studies show that objective well-being does not necessarily lead to subjective well-being (Kahneman and Deaton, 2010).

The role of religion and spirituality in fostering resilience has been extensively studied with regard to immigrants, primarily through quantitative methods (Amit and Riss, 2014; Formoso-Suárez et al., 2022; Klokgieters et al., 2019; Simkin, 2020). These studies have demonstrated that religion has the capacity to ensure resilience among immigrants. However, these studies often focus on religious affiliations in the host country and do not delve into the connection between religious practices adopted from the home country and immigrant experiences. Lusk et al. (2021) highlighted that despite high levels of post-traumatic stress, migrants score high on measures of resilience and quality of life, primarily due to their high levels of religiosity. Sanchez et al. (2019) found that religious institutions can be a significant source of support for recent immigrants struggling to adapt to a new culture. Social capital associated with religious involvement has also been identified as a crucial factor in reducing stress (Chen and Williams, 2016; Fry, 2023). These findings highlight the role of religion as a determinant in reducing stress among immigrants. Given that immigrants’ lives are more fragile compared to natives, especially when navigating a completely new environment, the support provided by religion is crucial in fostering hope (Vishkin and Ben-Nun Bloom, 2022). The spirituality of immigrants and their ties to religious institutions often help reducing acculturative stress. Revens et al. (2021) found that the resilience of immigrants is positively correlated with social support and religiosity. Some studies indicate that associations with religious institutions and spiritual experiences offer several benefits to immigrants, primarily helping them overcome psychological challenges in host societies (Abraham et al., 2022).

Furthermore, religiosity and social support can intersect with the cultural identity of immigrants (Revens et al., 2021; Schmees et al., 2024). As a result, one group of immigrants may be more resilient due to their religiosity, while another may not be. Research shows that Latin American immigrants in Europe and the Americas exhibit higher levels of psychological resilience due to their strong bonds with religion (Revens et al., 2021; Sanchez et al., 2019). However, research regarding Asian immigrants, who represent a large majority, is yet to emerge. While it is proven that religion can support resilience and increase subjective well-being, it is unclear how different groups of immigrants use religion to ensure subjective well-being. The use of religion by different immigrant groups can be unique, as their cultural roots are diverse. The appropriation of such cultural roots in determining the subjective well-being of immigrants is an area where research is still emerging.

The nexus between religion and migration has been studied from various perspective, with a primary focus on religion as a belief system that ensures spirituality and as an institution that supports social networking. For instance, Reyes-Espiritu (2023) revealed how labor migrants use faith as a coping mechanism during transnational mothering, suggesting that investing in faith could help them focus on their migration journeys and align with their aspirations. Morales et al. (2023) found that religion plays a crucial role in providing hope for immigrants, particularly in ensuring the safety of children and families. On the other hand, Behtoui (2022) and Gherghina and Plopeanu (2021) demonstrated that building social capital is closely related to religion. Udayanga (2024) and Udayanga et al. (2023) also discovered that religious institutions play a key role in facilitating immigrants to gather and discuss common concerns when constructing social networks. These institutions, which have connections to the home country, can effectively integrate dispersed immigrants into a common place. This was also observed by Moynihan and Glazer (1963), who suggested that immigrants tend to be less integrated culturally and strive to preserve and uphold their home country cultural beliefs. Therefore, hybrid identity of immigrants can influence how they navigate the host society.

Worldwide data show that religious people are generally happier compared to those who are not religious and have no religious affiliations (Lewis and Cruise, 2006). Research also shows that immigrants have a higher level of religiosity and higher levels of attendance in religious institutions when they have strong bonds to their home country (Gherghina and Plopeanu, 2021). Xu and Zheng (2023) discovered that voluntarism and philanthropic participation can also increase when religiosity is heightened. This was not merely a qualitative observation; several quantitative studies which use a large database of surveys also indicate that more religious people tend to be happier compared to those who are not religious (Steiner et al., 2020).

Religion has a significant impact on spiritual well-being, which is closely related to subjective well-being. The quality of life, or subjective well-being, is often closely associated with spiritual well-being (Simkin, 2020; Udayanga, 2020). Although difficult to define, spiritual well-being refers to an expanding sense of purpose and meaning in life, which can encompass one’s own morals and ethics (Whitford and Olver, 2012). Religion does not necessarily contribute to spiritual well-being, yet it is one of the most prominent drivers. The mental health of immigrants has received considerable attention, and research shows that spiritual coping can help immigrants achieve psychological stability (Eppsteiner and Hagan, 2016; Nordin and Otterbeck, 2023). Spiritual coping is closely associated with religion and cultural meanings, and the religion can be adopted as a mechanism for spiritual coping (Udayanga, 2020). For example, Agyekum and Newbold (2019) have shown that the socio-emotional and physical health of immigrants are significantly associated with spirituality that emerges from religious associations. Udayanga (2020) theorized that distress can be relieved, and psychological well-being can be stabilized through spiritual coping. However, culture based religious rituals play a crucial role in redefining the uncertainty of life episodes. Immigration, being an uncertain life episode, can thus call for religious coping. Yet, the ways in which immigrants reconfigure their home country’s religious practices in their host country remain unclear. Molli (2022) found that the gender dimension of religious experiences is critical, as immigrant women tend to be religious and are better able to maintain resilience even when emergencies occur, compared to those without religious affiliations. Her longitudinal qualitative study supports the idea that the role of religion, particularly for immigrant women, is paramount in promoting mental well-being.

## 3 The case of Italy

Italy has a historical record of welcoming immigrants, which prominently includes family migrants, labor migrants, and refugees (OECD/European Commission, 2023; OECD, 2023b). As a country with a strong religious presence, religion can play a crucial role in increasing happiness among immigrants in Italy (Ambrosini et al., 2021). Italy has become a major destination for immigrants, with a strong emphasis on labor and family migration, supported by policies aimed at better integration (Ambrosini, 2001). Compared to other European countries, Italy has a notably strong religious influence on immigrants. Laura (2020) also describes how religion is crucial for immigrants to integrate in Italy and to realize their expected well-being goals. Among the various immigrant groups, the present study focuses on Sri Lankan immigrants in Italy. The research was initially designed to explore the integration trajectories of South Asian immigrants, and the present paper is limited in providing an analysis of the happiness experiences of Sri Lankan immigrants who settled in Italy.

Migration has been a key driver of demographic and labor market dynamics in many migrant receiving countries in Europe, including Italy. Italy, along with other European countries, has hosted a large number of educated labor migrants and refugees (OECD, 2023a). Over time, migration has become a crucial force for demographic change and labor market needs. The rising ageing population has also compelled policymakers to invite a significant number of immigrants to Italy, both as laborers and permanent residents (Del Boca and Venturini, 2014). Sri Lankan immigrants in Italy represent one of the oldest immigrant groups, with migration to Italy starting as early as 1970 and experiencing a significant influx after 1980 (Bitonti et al., 2023). Italy has welcomed a large number of immigrants from Sri Lanka over the past decade. In 2022 alone, it received a total of 241,000 new immigrants on a long-term or permanent basis (OECD, 2023b). Many Sri Lankan migrants have chosen Italy as both a living and working destination (Henayaka-Lochbihler and Lambusta, 2004; Pathirage and Collyer, 2011). Several have decided to migrate with their families, and some have married natives in Italy. Although no studies have been conducted on emigration due to gender based violence, we have observed that several queer individuals are emigrating to Europe to express themselves freely and live the life they desire. Italy is one of the most attractive destinations for such individuals.

According to recent data, approximately 110,000 Sri Lankans reside in Italy, dispersed throughout the country, with a concentration in urban areas (Bitonti et al., 2023). Many have established permanent residency and actively encourage fellow Sri Lankans to migrate. Both men and women often find work in the healthcare sector, particularly Catholics. In recent years, there has been a notable influx of Catholic women employed as carers in nursing homes, contributing to the growing Sri Lankan community (Pathirage and Collyer, 2011). The exact number of Sri Lankans in Italy remains uncertain, partly due to illegal migration. However, they demonstrate a strong sense of community, supporting each other regardless of their arrival circumstances (Henayaka-Lochbihler and Lambusta, 2004). Some migrated during the Sri Lankan civil war, while others sought economic opportunities due to recent financial difficulties in the home country. Additionally, studies suggest that Catholic identity plays a significant role in choosing Italy as a destination (Pathirage and Collyer, 2011). These factors collectively contribute to growing presence of the Sri Lankan immigrants in Italy.

## 4 Theoretical framework

The subjective well-being model proposed by Durayappah (2011) has been adopted for this study, providing the basic framework for defining subjective well-being in terms of its experiential dimension. Therefore, the interviews primarily focused on the experiential nature of happiness and religious involvement. Given the interpretivist approach, we limit our study to understanding how immigrants perceive and make sense of their presence in respective religious communities. Durayappah’s (2011) model mainly emphasises life satisfaction, higher positive emotions, and lower negative emotions, which are also closely associated with religious affiliation, and spirituality.

Furthermore, we employed social network theory and social capital theory, as suggested by Vacchiano et al. (2024) to understand immigrants’ religious behavior in terms of their connection to religious experiences adopted from their home country and pursuit of happiness in the host country. Social network theory is an important perspective in migration studies, as social networks among migrants facilitate their life trajectories. Although social networking is a broad theoretical framework, our focus is on how productive interactions among Sri Lankan migrants contribute to a sense of community, emerging through religious engagement. Social networking theory is associated with the Settersten et al.’s (2024) theorization on unlinking and linking. As Settersten et al. (2024) have shown, linking and unlinking are significant life events that greatly impact one’s life course. They suggest that unlinking or separating from a group of people and linking with another can determine life transitions and status changes. We theorize that unlinking from the home country and connecting with a host country can shape the immigration trajectories of Sri Lankan individuals and influence how they navigate the host society. Unlinking from the home country compels immigrants to link with the host country, which can possibly modify the way in which they perceive their immigrant lives. During their life course, how home country based religious affiliations and beliefs emerge as supportive mechanisms for immigrants is given focus in the present paper.

We also employ Bourdieu’s (1977) social capital theory to explore bonding social capital or social networks within groups, which can foster beneficial affiliations that drive subjective well-being. Productive social relationships within the same immigrant groups are beneficial for resilience of immigrant lives. Settersten et al. (2024) further illustrate that unlinking is embedded in informal social processes. This is crucial for us to understand how unlinking is associated with religion as a cultural process. Although this theoretical paradigm does not explicitly address relinking, we theorize that relinking is closely associated with unlinking and provides individuals with a sense of community belonging. Relinking is the process of establishing connections with unlinked home country culture. The practice of continuing home country religious rituals indicates a virtual relinking to home country traditions. Cultural assimilation is a strong predictor of subjective well-being (Berry, 2005). However, the continuation of home country religious beliefs and rituals can indicate a reluctance to assimilate and a greater willingness to integrate while preserving their identity.

Religious pluralism is an important perspective in understanding the religious affiliations of immigrants, as it highlights how host-country religions become integrated into immigrant lives alongside the religious beliefs they bring from their home country. Religious pluralism refers to the coexistence of diverse religious beliefs, practices, and traditions, and societal responses to this diversity (Hick, 2010). Some argue that while several religions may coexist and individuals may engage with multiple belief systems, one religion often becomes dominant at a given time (Dein, 2020). This concept, we refer to as *ultimist religious pluralism*, suggests that a particular religious belief becomes prominent based on the believer’s intention and engagement (McEntee, 2017). This theoretical framework is important for understanding and formulating research questions on how immigrants practice and navigate multiple religious beliefs in host societies, alongside their migration aspirations.

## 5 Methods

Our study aimed to explore how a group of immigrants maintain their home country based religious practices as a means of spirituality and social networking for resilience. The exploratory nature of the research led us to employ a qualitative design, drawing on data gathered from participants (Bryman, 2008). Constructivism served as our ontological framework, recognising that reality is co-created through individual interactions and reflections (Crotty, 1998). Instead of concentrating on the evaluative dimension of subjective well-being, we focused on its experiential dimension, which is strongly influenced by cultural factors such as religion, among others. As immigrants navigate a new society and culture, they adapt their way of life to achieve their migration aspirations, incorporating both past and present experiences. This aligns with Durayappah’s (2011) theorisation that past experiences and future life prospects can influence an individual’s present experience of happiness. Therefore, understanding how they make sense of their new world is crucial for uncovering the complex links between migration and religion. Constructivism emphasises the importance of context, subjectivity, and individual perspectives (Bryman, 2008). Coupled with interpretivist epistemology, this approach allowed us to delve into the lived experiences of participants, exploring their interpretations of the migration-religion nexus. This method offers an avenue to focus on subjective interpretations and the co-constructed nature of reality.

Since this research is guided by interpretivism and constructivism, both participant selection and question formulation followed these epistemological assumptions. Interview questions were designed to explore how participants interpret their current lives, cultural connections, and religious beliefs. Participants were therefore encouraged to reflect deeply and provide detailed responses, revealing personal perspectives rooted in their community lives. Moreover, attention was given to understanding how past experiences and future prospects shape their present experiences related to religion and happiness.

We collected data from interviews with long-settled first generation Sri Lankan immigrants in Italy and conducted direct observations. Our focus was primarily on immigrants who have migrated for economic or family reasons, meaning they are voluntary migrants who mainly emigrated to improve their quality of life, which is difficult to achieve in their home country. Before constructing the interview guidelines, initial brief discussions were conducted with potential key informants. Participants were then recruited initially through purposive sampling, followed by snowball sampling. We adopted purposive sampling at the outset, as probability sampling was not feasible given the nature of the explorative nature of the study. While acknowledging that this sampling approach might introduce certain biases, our goal was not to generate multiple explanations for happiness. Instead, since the study directly explores how home country religious beliefs manifest among immigrants, purposive sampling was employed first, with subsequent participants recruited via snowball sampling. Community networking among immigrants is an informal social process; therefore, leveraging these informal networks and connections was beneficial and strengthened the intentional selection of participants.

We contacted potential Sri Lankan immigrants through their social networking groups and recruited initial participants who were settled in Italy. Directed by these participants, we selected other participants. Purposive sampling was crucial in our study, as our main goal was to explore how Sri Lankan immigrants engage in religious activities, primarily to ensure their migration aspirations, which ultimately lead to subjective well-being (Bryant and Charmaz, 2019). Informed by the first interviewed participants, we recruited all other participants for the study. Therefore, our study is limited to a certain Sri Lankan immigrant community networked through informal social connections. All immigrants came to Italy as family or economic migrants, and we did not focus on other types of migrants, which may be a future research focus.

We began data analysis early on and continued it phase by phase. Therefore, we employed the principle of theoretical saturation to terminate further sampling (Bryant and Charmaz, 2019). Even though we employed thematic analysis, we devised it iteratively such as in grounded theory. Therefore, theoretical saturation of sample was facilitated. In total, we interviewed nineteen participants, which included both men and women. All participants self-identified as either Buddhist or Catholic/Christian. All of them are associated with a Sri Lankan-origin religious institution in Italy. However, this does not necessarily mean that all Sri Lankan immigrants are associated with religious institutions. We focused on those who have religious affiliations with Buddhist background or Catholic Background, which is also a limitation of our research. Although Muslim and Hindu Sri Lankan communities are also present in Italy, we limited this study to two religious groups. A same study approach can be found in Laura (2020), where a study was conducted to understand how religious paths are associated with resilience focussing on Buddhist immigrants.

We initially conducted case studies with four religious leaders in Italy who had immigrated from Sri Lanka to establish religious institutions. These leaders included two Buddhist monks, one Catholic priest, and a local healer known for performing sorcery and witchcraft, practices that are quite popular among Sri Lankan immigrants in Italy. These case studies provided us with valuable insights into how Sri Lankans form and develop social networks based on religious institutions. They also offered rich data on how immigrants strive to maintain their home country’s religious values. The key informants shared how religion is reshaped in Italy to align with the destination culture and migration aspirations.

Following those key case studies, we observed ritual practices at selected two religious institutions. With the approval of incumbents, and of participants all observations were conducted. At a Buddhist temple, we observed a Sunday religious study class organized for Sri Lankan students. With permission, we stayed at the temple to observe how Sri Lankan children are taught and how they engage in various activities. In addition, another observation was conducted at a church where Sri Lankan Catholics get together and organize for charity event. We were also provided with videos of a religious celebration, which offered insights into how Sri Lankans come together to organize religious ceremonies. Subsequently, we conducted in-depth interviews with nineteen participants within their family settings. Thus, our interviews were primarily based on nineteen families, involving husbands, wives, and sometimes their children. In addition, we recruited participants in the working age, mainly between 25 to 50 years of age, who are also married and settled in Italy. Our focus was also on families rather than individuals. Although individual participants were selected through snowball sampling, the interviews were conducted within family settings, which often involved groups. Therefore, our focus was on families associated with religious institutions. This focus is also one of the limitations of our study, and hence families without non-religious background was not included. Table 1 summarizes the sample demographics.

**Table 1:** Participants’ demographic information.

No	Gender	Age	Years since migration	Religion
1. Case	Male	50	25	Buddhist
2. Case	Male	55	30	Buddhist
3. Case	Male	43	10	Catholic
4. Case	Male	50	16	Buddhist/Healer
In-depth interviews				
5	Female	31	10	Catholic
6	Male	26	20	Catholic
7	Male	42	14	Buddhist
8	Male	43	26	Buddhist
9	Female	43	23	Catholic
10	Male	40	17	Buddhist
11	Female	31	14	Catholic
12	Female	36	9	Catholic
13	Male	33	11	Buddhist
14	Female	50	26	Buddhist
15	Male	39	14	Catholic
16	Female	40	19	Buddhist
17	Female	46	21	Buddhist
18	Female	41	16	Catholic
19	Male	36	16	Catholic
20	Female	38	18	Catholic
21	Male	43	19	Buddhist
22	Female	44	28	Buddhist
23	Male	46	21	Buddhist

We used a semi-structured interview guideline to collect data from participants. These focused areas were mainly guided by our epistemological assumptions. The questions primarily focused on (1) the challenges they encountered as individuals and family members while navigating the host society environment, (2) the religious affiliations and beliefs they adhere to, (3) ritual practices and witchcraft, (4) reasons for practicing witchcraft and sorcery, and (5) reasons for continuing home country religious practices and their tendency to assimilate into the host society. Voluntary participation was guaranteed, with participants having the option to withdraw at any time during the interview. Informed consent for participation was obtained, and observations in some religious institutions were permitted by the incumbent monks. In-depth interviews lasted 90 to 120 min per session. With the participants’ consent, fifteen interviews were recorded. Only four interviews were handwritten. Additionally, important events of religious institutions and families were video recorded and photographed. Previously taken photographs were also obtained from some selected participants with their consent for publication where necessary. For the sake of anonymity, narrative excerpts presented in this paper do not include personal identifiers. All data collected are stored in a secure computer, where access is granted only for researchers. The identifiable faces of people appearing in photographs were blurred, only to capture the incident.

Thematic analysis is generally conducted at the conclusion of data collection (Braun and Clarke, 2019), but we began analysing data at the inception of data collection. As a result, our analysis was reflective and iterative. We chose to analyze data iteratively from the beginning of data collection because it helped us determine theoretical saturation and was time-efficient, preventing data redundancy. Initially, we identified five themes and collected data specifically focusing on them. However, during the second stage of analysis, we realized these themes could be grouped into two broader categories. As a result, data collection was guided by both the five sub-themes and the two broader themes. Data saturation was determined collectively by the researchers through an iterative process of data analysis and reflection. Through iterative analysis and reflective engagement with the data, we determined that the five sub-themes had reached saturation and could be grouped into two broader themes. No additional insights or directions emerged from participants.

First, we listened to recordings and reviewed field notes after every interview or set of few interviews, transcribing them verbatim. Observations from selected religious events were also described, and written notes were provided for a holistic understanding and integration with interview transcriptions. The analysis was inductive in nature. Reflexive thematic analysis was important because it allowed us to engage reflectively and thoughtfully with the data throughout the analytical process. Therefore, our experiences and reflections influenced the process of developing themes. As suggested by Braun and Clarke (2019), we focused on latent coding, which allowed us to go beyond the descriptive level of data and identify deep meanings, ideologies, and patterns in the data.

The first three steps of thematic analysis (familiarising ourselves with data, generating initial codes, and searching for themes) were performed at the initial stage, and thereafter, we reviewed emerging themes at every stage. We used MAXQDA software (2020 version) for data analysis. After each interview and the inclusion of observation data, we followed the first three steps of thematic analysis and reviewed generating themes. We employed line-by-line coding, focusing particularly on the latent and apparent meanings provided by participants. Theory driven codes were also identified, and subsequently, all codes were matched to generate initial draft themes. Initially, we developed five themes, but over time, all themes were refined into two coherent themes that describe how religion and spirituality intersect with immigrant lives and their possible connection to subjective well-being. Finally, all identified stories were gathered around two themes, and example excerpts were provided to create a coherent theoretical narrative.

We adhered to quality criteria proposed by Tracy (2010) to ensure the trustworthiness of our study, and we used consolidated criteria for reporting qualitative research in our reporting process (Tong et al., 2007). As Tracy (2010) outlined, the quality of qualitative research begins with selecting a worthy topic for qualitative inquiry, which aligns with our research. The credibility of the study was established by conducting the research iteratively, keeping participants informed, and incorporating their reflections before proceeding to subsequent steps. To ensure transferability, we provided clarifying notes at each stage of data collection and analysis, similar to memos used in grounded theory. Furthermore, collective data analysis supported the credibility and transferability of our findings. Member checking was also implemented to verify whether analytical narratives accurately reflected participants’ experiences.

Reflecting on our interviews with key informants (case studies), we found that they were community members with extensive experience in the migration-religion nexus over a long period of time. They understood how immigrants interpret their migration trajectories in line with religious engagements. Thus, key informants spoke not only for themselves but also for the immigrants who participate in religious activities. Since in-depth interviews were conducted in the presence of family members, participants discussed their religious experiences as part of a collective meaning-making process. These interviews provided participants with an opportunity to reflect on their immigration trajectories as influenced by religious affiliations and spirituality. Using multiple data collection methods allowed us to cross-validate our findings through triangulation. In addition, peer debriefing was conducted, in which two experts in the study area reviewed the research process at various stages, encouraging us to consider alternative perspectives.

Furthermore, the personality characteristics of the researchers, such as gender and age, can impact the study. The fact that one of us is male and the other female enabled us to interview participants within their families comfortably. One of us being a Buddhist and the other a Catholic also allowed us to understand the meanings revealed by participants as clearly as possible. These are the strengths of our study, as two different religious affiliations enabled us to fit into two major religious settings. One of us has experience living in Italy, which was a crucial factor in recruiting participants and understanding immigrant behaviors related to the destination country. To reduce researcher bias in the study, we employed several strategies. We regularly reflected on personal values, beliefs, and assumptions, maintaining a reflexive journal throughout the research process.

### 5.1 Findings

**Figure 1:**
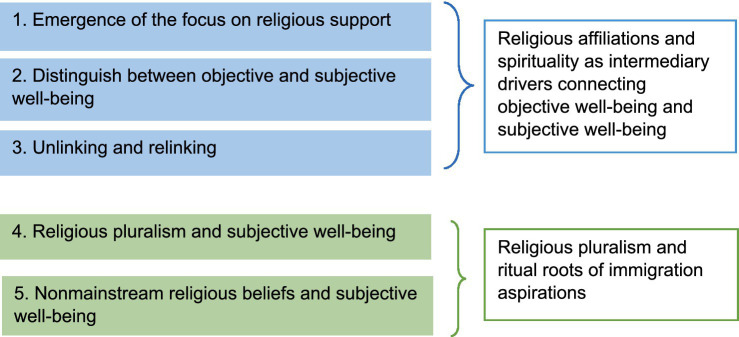
Thematic map. Five sub themes are categorized into two main themes.

Participants consistently indicated that, in culturally diverse social settings, maintaining a strong sense of belonging to their home country’s (even virtually) can help them manage uncertainties through spiritual practices and religious engagement. We found that religious practices and participation in religious institutions have adapted to the Italian social context while continuing to uphold values associated with the home country. Although several symbolic meanings attached to religion have shifted, exploring those details is beyond the scope of this paper. Instead, we focus on how these reconfigured home country based religious institutions facilitate establishing subjective well-being through the two identified themes. Figure 1 shows the thematic map.

### 5.2 Religious affiliations and spirituality as intermediary drivers connecting objective well-being and subjective well-being

#### 5.2.1 Emergence of the focus on religious support

Sri Lankans migrating to Italy often do so either to escape conditions in Sri Lanka that negatively affect their quality of life, or to enhance their existing well-being. Highly educated individuals typically migrate seeking improved opportunities to ultimately raise their standard of living. Participants in our study reported successfully securing jobs with significantly higher wages compared to Sri Lanka and establishing stable family lives in Italy. Even participants who undertook less skilled work, which was unavailable in Sri Lanka, generated sufficient income to support their long term integration. These factors notably increased immigrants’ happiness, particularly during the early stages of migration. This increased well-being was often accompanied by feelings of sadness for those left behind in their home country.

“It saddens me to think of the people in Sri Lanka when I see the way people live in Italy. I know that this would be a dream for many Sri Lankans. Our countries might take at least a millennium to reach this level of development”. (Woman, 31-years-old)

“Even working as a cashier is considered good here. Nobody judges your status based on your job. If I did this job in Sri Lanka, I would definitely be regarded as a lower class person. And wages there are very low. Here, I can earn as much as I am able to, and it far exceeds even a well-paid job in Sri Lanka”. (Man, 26-years-old)

Immigrants initially connect to the host society primarily through integration into the labor market, and this generally enhances their subjective well-being. Their positive feelings increase due to objective success, especially when compared to their situation in Sri Lanka, where limited success generated negative feelings. They virtually conceptualize what their lives would have been like had they not migrated, and this comparison reduces negative feelings on the current lives, thus increasing their relative happiness.

Initially, the objective well-being that immigrants attain helps them achieve greater life satisfaction, thus contributing to subjective well-being. Their connection to the new host country, combined with comparisons to the life circumstances they might have faced in Sri Lanka, generally enhances their overall life satisfaction. At this stage, the role of religion or spirituality is less evident. When immigrants successfully fulfil their primary migration aspirations, they tend to experience increased happiness—a finding supported by other quantitative studies as well. Using their home country as a reference point further heightens this sense of happiness, again with no clear influence of religion. As a result, newcomers are less likely to prioritize religion or spirituality initially, as their primary focus remains on establishing objective well-being.

On the other hand, when achieving objective well-being appears challenging, immigrants tend to seek assistance from supernatural or spiritual entities, often facilitated through religion. The uncertainties associated with migration journeys can be partly alleviated through involvement with religious institutions and engagement with spirituality.

“I had to stay on the streets for about seven days without a proper place to stay. I thought that I had made a bad decision to come here. However, at that time, I happened to meet a priest who runs a temple for Sri Lankans. He helped me find a job. I thanked God for leading me to such a person”. (Man, 42-years-old)

The uncertainty of immigrant journeys is common for some immigrants. They can resolve challenges encountered through various means, yet spirituality and religious affiliation can better facilitate overcoming these uncertainties while stabilising psychological balance. Therefore, at the beginning of migration journeys, many immigrants are less likely to focus on religion or spirituality as drivers of happiness. They only turn their focus to religion or spirituality when they encounter uncertainties. They seem to believe that any supramundane power would help them overcome challenges, which motivates them to affiliate with religious institutions. Often, based on previous experiences, they choose religious institutions from their home country, as they believe these institutions are there to help Sri Lankans.

“Many participants are settled Sri Lankan immigrants who engage in religious activities. Occasionally, some newcomers join in, but they do not consistently participate in these activities.” (Case 3)

“Newcomers and young people turn to us when something bad happens to them or when they anticipate that something bad might occur.” (Case 4)

Immigrants face a great burden, as they often feel like outsiders in their new country and seek spiritual support and social networking. This predicts immigrants’ religious affiliations with their home country. Immigrants often turn to the religions of their home country because these beliefs are ingrained in their cultural upbringing, and they are well aware of how these religions can be utilized for blessings and ensuring future stability. Some Catholic immigrants do attend religious establishments in Italy because they believe Italy to be the original centre of Catholicism, though this is not the case for Buddhist immigrants. While spiritual support is sometimes sought from Italian religious institutions, many immigrants prefer to attend Sri Lankan religious organizations in Italy. They find that meeting fellow Sri Lankans in these institutions contributes to their sense of community and belonging. A 43 years-old man who immigrated fifteen years ago elaborate:

“We do, in fact, engage in different religious activities at Italian churches since we think these possess some healing ability. Simultaneously, we often go to events hosted by fellow Sri Lankans where we meet like-minded individuals with whom we may exchange ideas. Although Italians follow our religion, their perspective differs from ours.”

Sri Lankan immigrants from a Catholic background actively participate in Italian religious institutions, viewing them as original centres of Catholicism. However, they often differentiate these from religious establishments originating from Sri Lanka, where they can interact with like-minded people. This highlights a strong inclination to maintain connections with their country of origin. Importantly, immigrants emphasize that although they integrate well with local Italians, interactions with fellow Sri Lankan immigrants offer greater comfort by enabling them to share thoughts and concerns common to immigrant experiences. Thus, social networking with culturally similar individuals through religious organizations can foster positive feelings and happiness. The importance of social networks and close social relationships as drivers of happiness is well established. We found that for immigrants, social networking occurs primarily through religious institutions, which serve as central locations for gathering. Even though several other social gathering places exist, religious organizations play a crucial role in facilitating gatherings because immigrants often view attending them as an obligation (that fosters collective consciousness among participants).

#### 5.2.2 Distinguish between objective and subjective well-being

Achieving objective well-being can enhance happiness in the first few years after immigrating to Italy. However, as immigrants gradually integrate into their new environment and begin to lead a settled family life, they start to distinguish between objective well-being and subjective well-being. Over time, objective well-being seems to diverge from happiness, and immigrants may feel a sense of worthlessness in carrying out activities associated with immigrant life. Some longitudinal studies suggest that while immigrants tend to be happier initially, they may experience feelings of worthlessness over time. We found that religion serves to restore a sense of purpose in life and balance overall happiness. Once their aspirations are fulfilled, the initial high levels of happiness begin to plateau, often replaced by a sense of hopelessness. This transition is often challenging for immigrants to articulate.

“We came and settled here. It was a struggle at the beginning, but we were happy because we believed that we had achieved something. However, over time, as I worked and interacted with the natives, I began to feel that, despite having money and freedom, something was missing. There was a void in my mind and life.” (Woman, 43-years-old)

“At the beginning, I felt alive. But after a few years, I found myself asking, “What now?”” (Man, 40-years-old)

Once initial aspirations are fulfilled, immigrants strive to seek a deeper meaning of life beyond mere objective well-being. However, at this juncture, objective well-being alone cannot generate subjective well-being. When the reference point shifts from the home country to the host country, objective well-being cannot ensure life satisfaction, because comparison with natives often generate a relative lack in life satisfaction. Loneliness can also erode happiness over time, even though a solitary life was initially identified as a factor that increased objective well-being for some participants. Building a family emerges as a solution to loneliness, and it is closely linked to religious affiliations and spirituality. Immigrants from Sri Lanka are likely to be more spiritual and affiliated with religious institutions, providing them with psychological stability and assurance in their future lives.

“I thought of having a family life because I was reminded of my parents advising me to care for others. This is instilled in me even when I see religious teachings.” (Woman, 36-years-old)

The stability of family life can be more fragile in a new cultural context, as evidenced by some participants who have experienced divorce. However, religious affiliations seem to stabilize family life in the host country. Although it may not be apparent, the collective religious affiliations can lead family members to focus more on the impact of strong family ties on their happiness. Sri Lanka is a country with strong family ties, and the same is expected in Italy, although it may be more fragile. This stability seems to be ensured when immigrants negotiate their family lives in line with religious affiliations, where family members participate together in religious observances and connect with their experiences in their home country.

“Alcohol addiction is quite common among many immigrants, with a significant number of them smoking heavily. I believe this is primarily due to their stress and feelings of hopelessness. Some approach us to discuss their lives, and from these conversations, I have come to understand that the lives of immigrants are extremely fragile. Without psychological stability, they cannot thrive. Those who are active in religious activities tend to thrive more than those who are not.” (Case 1)

Although objective well-being can bring happiness to immigrants initially, it gradually became disentangled from subjective well-being, leading to a void. This void compels people to consider several mechanisms to ensure happiness, including building a family life or engaging in volunteerism. We found that association with religious institutions can reconnect immigrants with like-minded people and link them to cultural experiences from their home country, ensuring that they extend their objective well-being into a wider community. This, in a way, increases their happiness. The collective meaning-making facilitated by religious institutions is an important driver of happiness.

“I realised that success shouldn’t be a solitary pursuit. There are many people all around me, so I must take them into account. I have a responsibility towards them. This understanding was instilled in me when I worked with people in the church and interacted with other Sri Lankans. I may not have remitted sufficiently to my parents back home, but now I think about them more carefully.” (Woman, 31-years-old)

Spirituality develops when the meaning of life is understood in the company of like-minded people, and immigrants often need support through religious practices and observances for this development. Collective volunteerism is also linked to religious activities, which help immigrants build social networks and foster a sense of belonging. The solitude of an immigrant’s life can lead to unhappiness, but this can be mitigated through religious affiliations because they facilitate meaningful social connections. However, merely having social connections is not enough; these connections must provide positive support for individuals. When immigrants invest in positive social networks, objective well-being can effectively connect with happiness.

“We also attend churches in this country, but we mostly prefer to go to places where Sri Lankans gather. In those places, we feel at home.” (Man, 33-years-old)

“When we go to the temple, we are reminded of why we came here. I then realised that many Sri Lankans came here for a purpose, and I must focus on that aspiration. This extends beyond Italy and involves helping those in Sri Lanka.” (Woman, 50-years-old)

The religion practiced by immigrants in their home country holds significance for our participants, as it reminds them of the aspirations they had before coming to Italy and aligns them with a strong hope for success. This suggests that religious affiliations virtually reconnect people to their home country, shifting the reference point for comparison from the host country to the home country. As a result, perceived happiness becomes linked to present objective well-being.

“The priest always reminds us that we come from a developing country, encouraging us to bring real-life experiences from Sri Lanka and compare them with our lives here. This motivates me to reconsider my definition of success and fills me with hope.” (Man, 39-years-old)

Some priests do not reside permanently in Italy; instead, they return to Sri Lanka periodically. These mobile priests play a crucial role in sharing real-life experiences from Sri Lanka with immigrants. This allows the immigrants to compare their current success with the situation in their home country, which significantly increases their happiness by validating that their decision to migrate was worthwhile. Moreover, when these immigrants attend various religious events with other Sri Lankans who share similar lifestyles and life goals, it provides a common platform for them to exchange experiences and ensure the generalization of these experiences. This generalization informs immigrants that the challenges they encounter are not unique, but rather common for all immigrants. This understanding helps alleviate the stress associated with their immigration trajectories.

“Once I attended the temple, I realised that I was not alone. It was not just my family; several others were also encountering similar or even worse problems. I could understand their experiences and share my own with them to find common solutions. Amidst a busy life, the temple is the only place where we can meet together.” (Woman, 40-years-old)

Both religious commitments and large-scale community gatherings—often organized by religious institutions—support the generalization of experiences. This leads to stability, making immigrants feel that they are not alone but part of a group of people who are likely to provide support in overcoming problems.

#### 5.2.3 Unlinking and relinking

Moreover, once immigrants unlink from their home country, they are less likely to feel a sense of cultural detachment immediately. However, over time, they may start to feel disconnected from the cultural life that once provided them with a sense of hope and meaning. Religion can reconnect immigrants to their home country’s culture through participation in religious and cultural activities. This process of disconnecting and reconnecting is a crucial dynamic for the subjective well-being of immigrants. Initially, disconnecting from Sri Lankan culture provides the impetus to create new experiences in Italy and motivates them to integrate through hard work. However, once they secure their objective well-being over time, the need to reconnect with their home country’s way of life reemerges, leading to feelings of not being fully assimilated into their new location. At this juncture, religion provides a virtual reconnection to the home country experience through the collective performance of several rituals.

For example, first-generation immigrants in our study often align their subjective well-being with Sri Lankan culture. Therefore, when living with their children, these parents expect their children to adhere to a Sri Lankan value system that promotes intimate relationships between parents and children. This is most achievable when children are exposed to Sri Lankan culture, which is primarily available in religious places such as Buddhist temples and churches established by Sri Lankan congregations. Intergenerational conflicts often arise as second generation immigrants adopt Italian culture, which is contrary to the expectations of their Sri Lankan parents. This discrepancy also illustrates why objective well-being does not necessarily translate into subjective well-being. The expectations that parents have for their children are less likely to be met without aligning the children with the Sri Lankan value system. Therefore, attending religious institutions can promote the Sri Lankan value system to some extent among children. This can provide them with a considerable Sri Lankan experience, such as learning Sri Lankan languages in religious institutions and celebrating Sri Lankan religious events.

“I worry when my child frequently goes out with other Italian children. In today’s world, diverse sexual orientations are more visible, and I fear my child might identify as gay. I have no objections to it, but I would prefer my child not to be gay. Therefore, I accompany him to the church and let him interact with Sri Lankan Catholics so he can understand our way of life and our values.” (Woman, 46-years-old)

“In this country, children do not seem to have very intimate relationships with their parents. However, we had close relationships with our own parents. We should impart these values to our children because they could support them in leading a good and virtuous life.” (Case 2)

**Figure 2:**
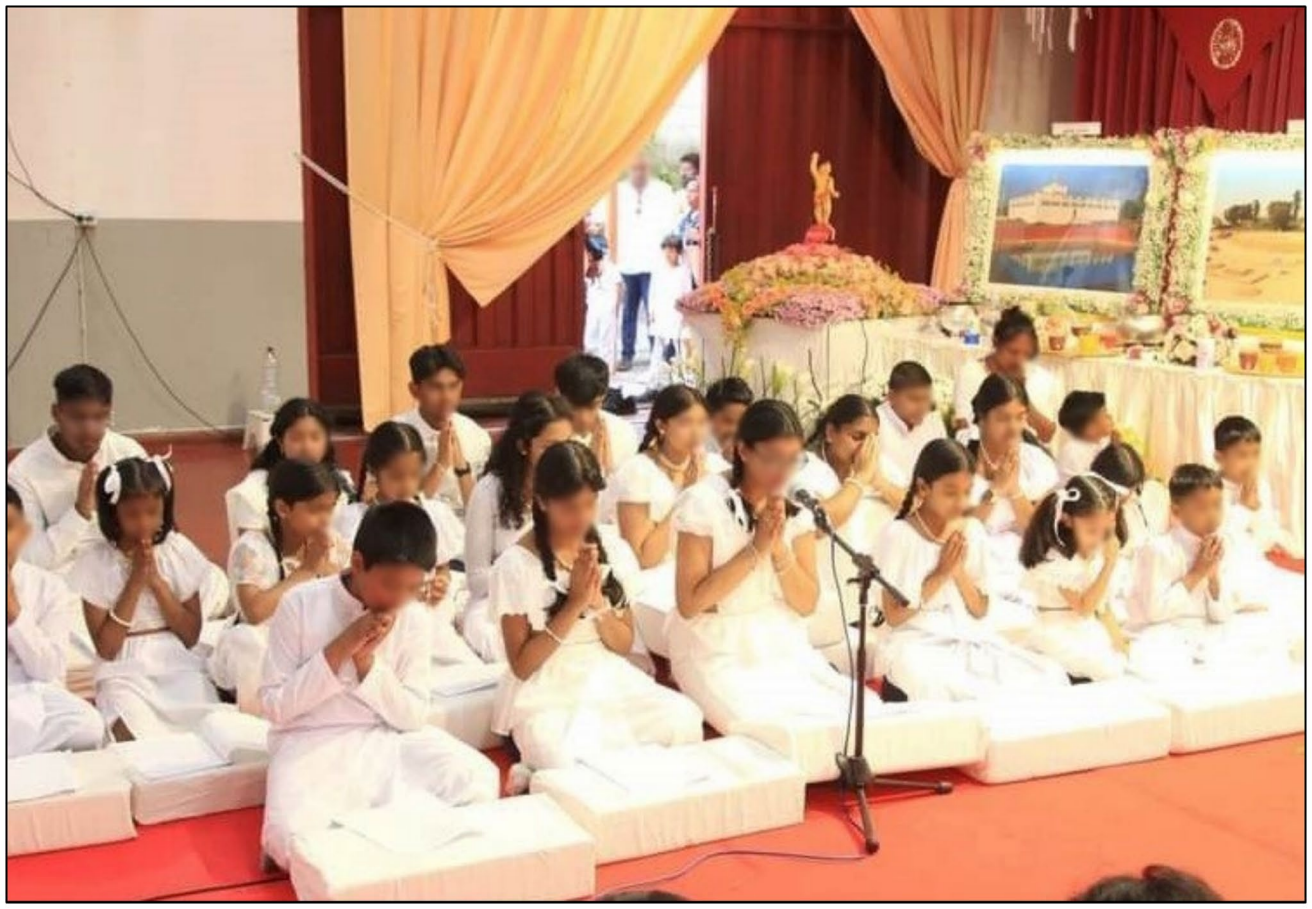
Sri Lankan children attending Sunday Dhamma school at a Buddhist temple. Buddhists generally send their children to Sunday religious schools. This practice has been promoted in Italy, with the belief that it helps children develop Buddhist values for a successful life. Source: Authors.

Sunday Dhamma schools for Sri Lankan children are important events that instil Buddhist values among them (Figure 2). One might question the purpose of sending children to religious institutions to learn Sri Lankan culture and values. After all, are immigrants not supposed to leave the past behind and embrace a new cultural life? However, even second generation immigrants are motivated to experience Sri Lankan culture. They attend religious study classes where they learn the Sinhalese language and Buddhist ethics and participate in organising Sri Lankan cultural events. Parents often encourage their children to learn Sri Lankan values, believing that it will lead to increase in happiness. The objective well-being achieved in Italy can only be complemented with subjective well-being when they incorporate the Sri Lankan value system, as they believe. This is facilitated by religious institutions and the close relationships immigrants form at religious gatherings.

Collective volunteerism, facilitated through participation in religious institutions, is another driver that enhances subjective well-being. Immigrants collectively encourage actions like giving to those in need, helping their home country, and enrolling children in religious value study classes. This platform is enabled by religious leaders, including Buddhist monks and Catholic priests.

“‘*We Sri Lankan Italians*’ is a group of Sri Lankan immigrants. We convene monthly at a chosen temple. There, we organise voluntary activities and plan programmes to support various Sri Lankan initiatives. In addition, we arrange different religious events each month.” (Case 4)

**Figure 3:**
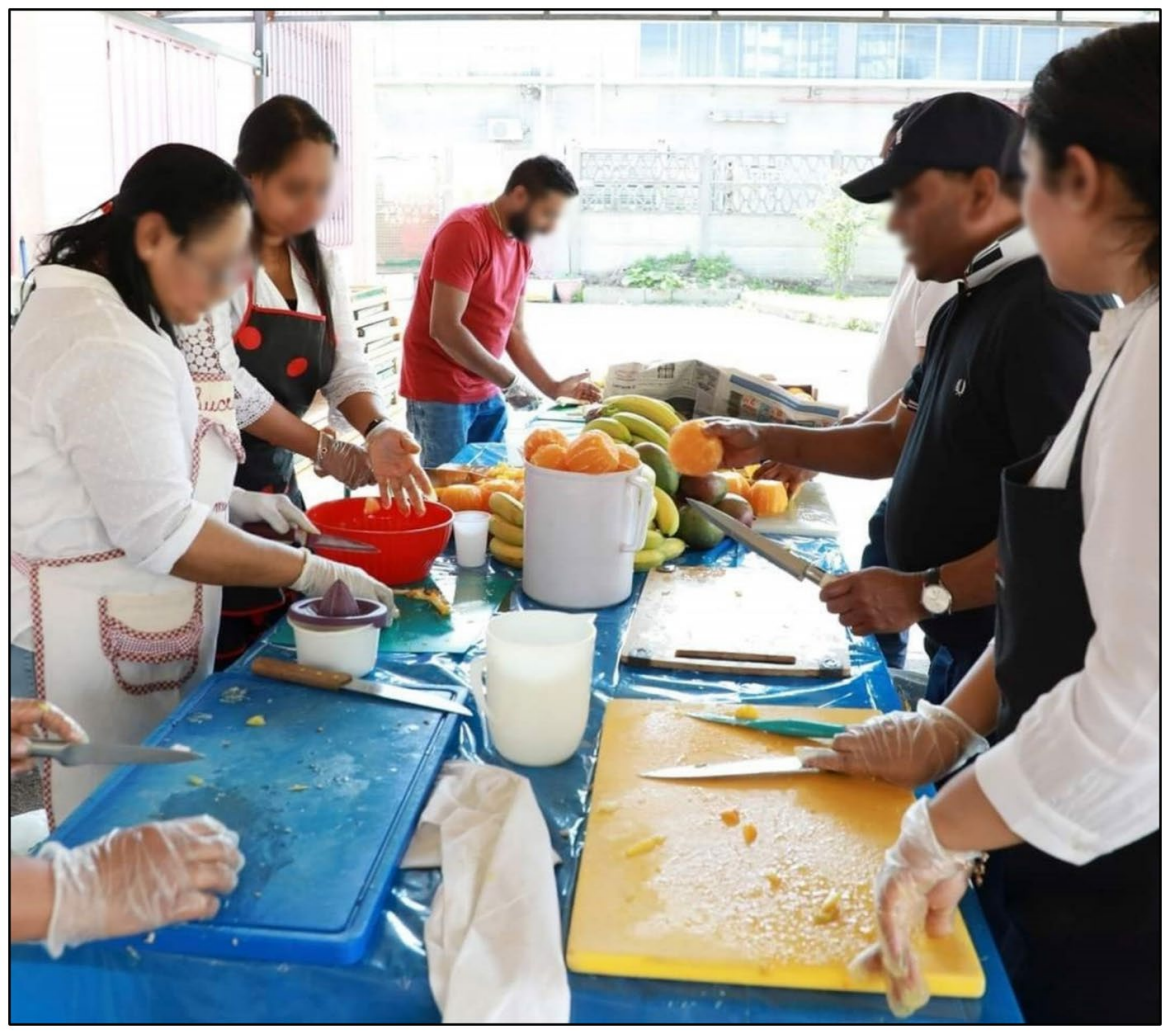
Voluntary activities (Preparing for a free meal giving event). An immigrant Buddhist community preparing a meal for a free meal-giving activity, fostering a sense of belonging within a like-minded community. Source: Authors.

The solitude that emerges in the new country can be mitigated through such voluntary activities (Figure 3). As participants described, it provides a sense of satisfaction in their lives as *immigrants* because they are now connected to other people. Religion is thus utilized not only as a spiritual path but also as a social path that links people to a broader community of like minded individuals. This connection also ties immigrants to Sri Lankan experiences, helping them feel at home. Therefore, this religious linkage is a crucial element that intertwines both objective well-being and subjective well-being.

### 5.3 Religious pluralism and ritual roots of immigration aspirations

#### 5.3.1 Religious pluralism and subjective well-being

We observed a pluralistic approach to religious practice, meaning that immigrants often do not confine themselves to a single belief system. Instead, they adhere to multiple belief systems, each fulfilling different needs. While they may have a primary belief system based on their upbringing, their immigrant identity encourages them to engage with diverse religious practices as part of their strategies for successful integration. This seemingly contradicts the traditional definition of belonging to one religion, but in reality, immigrants often integrate multiple belief systems at the spiritual level. Potential future uncertainties and anxieties are the driving force behind this pluralistic approach, which may lead people to seek solace and support from various religious sources. This may even include local beliefs associated with witchcraft or sorcery, further highlighting the complexity and diversity of immigrants’ spiritual engagement.

**Figure 4:**
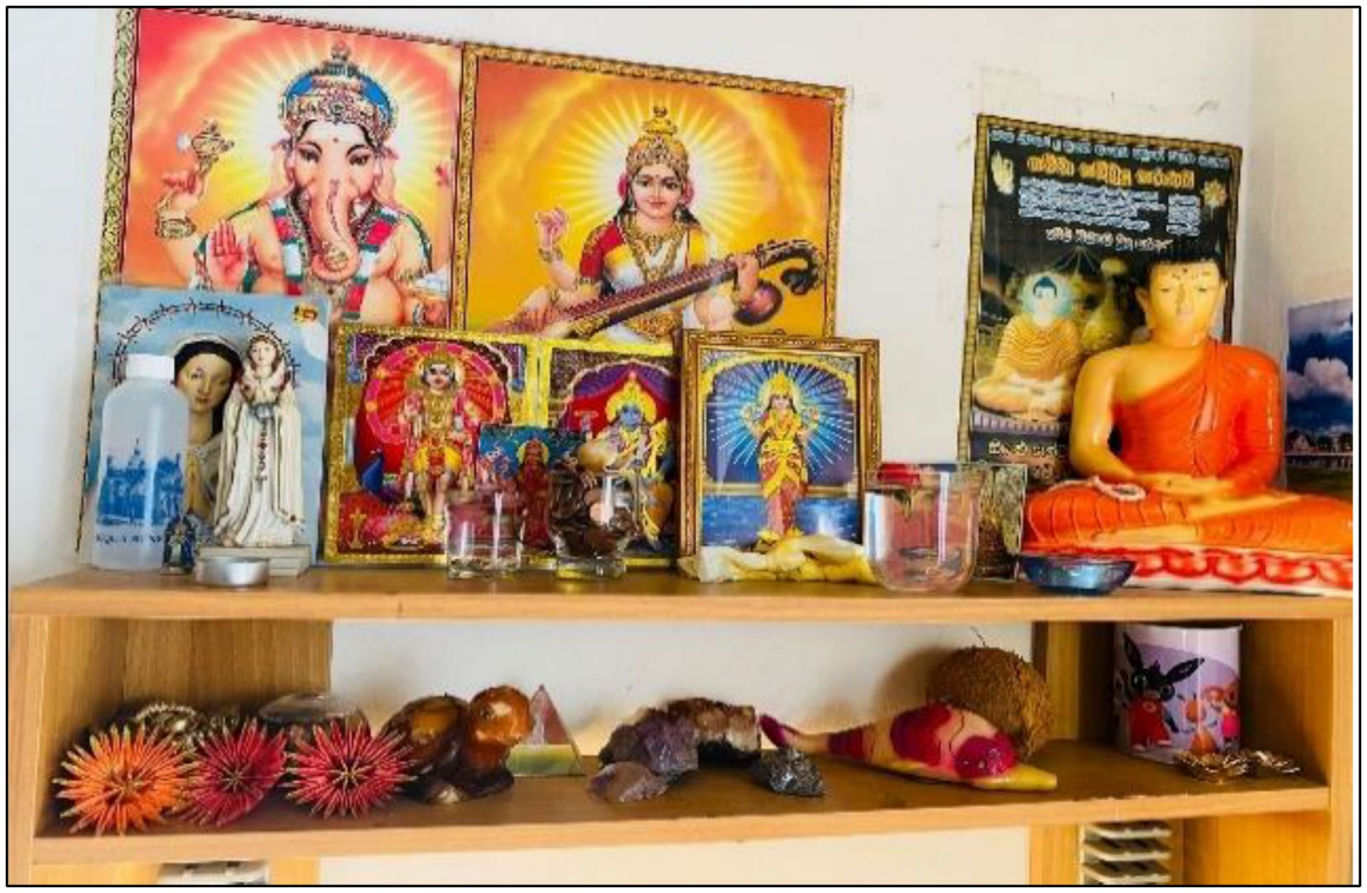
Worshiping several idols belong to different religions. This figure shows how different idols coming from different religious traditions are placed in the same location. Source: Authors.

In some families, we observed idols from different belief systems placed together and worshipped for blessings (Figure 4). Immigrants in these families explain that they worship multiple gods to receive blessings from various sources, with the hope that this will ensure their lives are secure.

Psychologically, the belief in protection from the universe can provide them with peace of mind, as evidenced by several studies (Ambrosini et al., 2021; Udayanga, 2021). Compared to native populations, immigrants often face greater social, cultural, economic, and political instability. The pluralistic nature of religiosity, to some extent, helps them address these challenges.

“We believe that we are governed by the universe. To us, God represents the universe. Therefore, we must align ourselves with the universal order. After all, all religions share common principles. Consequently, we believe that every deity will guide us towards happiness if we adhere to the virtues they embody.” (Man, 36-years-old)

Perceived uncertainty is an abstract concept that may manifest in the future. However, as immigrants, Sri Lankans in Italy are cautious about future events and strive to prepare for them, even using religion as a cornerstone. This perceived uncertainty can lead to pluralistic religious behavior. Moreover, the worship of several gods representing their country of origin helps reconnect immigrants to the experiences of their home country (see Figure 4). On the other hand, real uncertainties in immigrants’ lives, including job loss and family separations, also lead them to seek help from multiple sources, including various religious beliefs.

“I wanted to bring my husband here because I knew that once I migrated, he would seek the company of other women in Sri Lanka. My mind was in turmoil here. That is why I sought the help of a person who performs witchcraft. They assisted me in performing a ritual to change my husband’s mind about coming here. It really happened, yet I do not understand the logic behind it.” (Woman, 41-years-old)

Believing in multiple religions simultaneously is a result of the perceived support that various belief systems provide for achieving various immigration aspirations. For instance, Buddhism may not assist them in achieving some mundane goals, such as a good marital life or warding off the influence of jealousy from others. Therefore, they adhere to other belief systems, including practices of sorcery and witchcraft, to achieve such objectives. Simultaneously, the lack of access to Buddhist institutions can encourage attendance at Christian religious institutions in Italy, as suggested by our respondents.

“Although I am Buddhist, it does not prevent me from participating in church activities. I attend these events to meet other Sri Lankans and to support people in organising charity and other volunteer activities.” (Man, 40-years-old)

“I live in Brescia, a city dedicated to the Catholic ‘Mother of Roses’. There’s a belief that placing a statue of the ‘Mother of Roses’ in one’s home brings blessings. We worship both the Buddha and the ‘Mother of Roses’. Interestingly, I secured a job after placing a statue of the ‘Mother of Roses’ in my home.” (Woman, 38-years-old)

**Figure 5:**
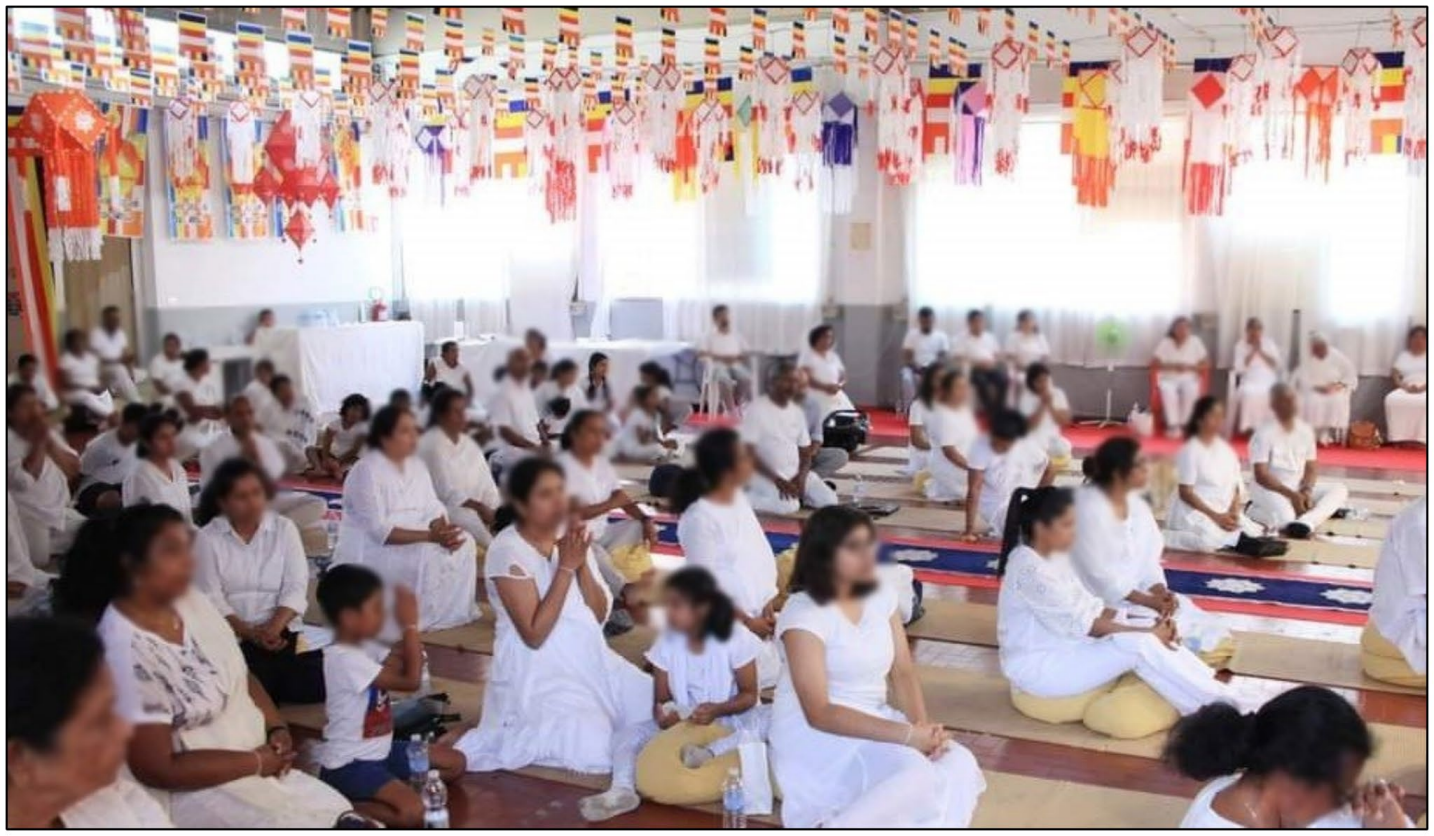
Religious gathering and practising Buddhism. Buddhists generally gather on full moon Poya days to observe Buddhist principles, fostering a sense of spirituality. Source: Authors.

Our analysis reveals that immigrants participate in collective religious celebrations in religious institutions, contributing to the cohesion of Sri Lankan immigrants. This participation fosters the development of an immigrant milieu where social capital is greatly enhanced. Religious social milieus, grounded in traditions from their home country, serve as one of the key drivers of immigrants’ happiness. These milieus act as shields, protecting immigrants from the uncertainties associated with their lives. Religious milieus are developed when experiences of immigrants are commonly shared and develop a feeling of belonging to a common group. Figure 5 illustrates a religious gathering of Sri Lankan immigrants in Italy, which has fostered in creating a strong social network.

The religious life of immigrants is a blend of various religious and cultural performances. Celebrations like Poya Day and participation in other religious and cultural activities provide immigrants with symbolic experiences that connect them to their country of origin. The religions practiced in the home country are significant because the aspirations for immigration, developed before migration, are often linked to Sri Lankan cultural experiences. Therefore, incorporating Sri Lankan experiences into a cultural framework is crucial for immigrants. It enables them to reflect on how they have developed their migration aspirations. Immigrants often find greater happiness due to their spirituality and religious affiliations, which virtually connect them to their country of origin. The values brought from Sri Lanka can be integrated with the Italian approach to objective well-being through religion, leading to an increased level of happiness.

During their immigration journey, immigrants may develop certain negative attitudes over time. These attitudes, often associated with the distress of acculturation, can lead to psychological instability and decreased life satisfaction. However, these self-generated negative attitudes can be transformed into positive ones when immigrants engage in religious practices. For instance, respondents indicated that acculturative distress, which stems from the gap between immigrants’ cultural lives and the prevailing Italian culture, could be alleviated by adhering to multiple belief systems. This approach might facilitate their integration into Italian society while preserving the values they brought from their country of origin. For example, a 43-years-old man describes,

“First, I came to Germany, which was a difficult time for me. Later, I moved to Italy, but the place where I live is also challenging. Understanding the way the natives think has been difficult, constantly reminding me of my life in Sri Lanka. It was then that I met some Sri Lankan immigrants who introduced me to church activities. Through them, I realised that I needed to revise my way of thinking and embrace the present.”

#### 5.3.2 Nonmainstream religious beliefs and subjective well-being

Sri Lanka is known for its widespread use of white and black magic, often employed to achieve everyday success. While the majority of Sri Lankans officially identify as Buddhist or Christian, many also incorporate white magic practices into their lives, seeking additional assurance of happiness. Notably, both Buddhism and Christianity condemn black magic, which is considered harmful to others. When individuals desire supernatural aid for such harmful purposes, they turn to traditional Sri Lankan witchcraft and sorcery. These practices are prevalent among Sri Lankan immigrants in Italy, where the fragility of their lives and interpersonal conflicts often lead them to seek support from these local belief systems. One immigrant describes:

“Occasionally, we consult a ‘*gurunnanse’* (sorcerer) for assistance when we encounter difficulties in our lives. We believe that many misfortunes occur due to the ‘evil eye’ of envious individuals. As per our belief, these jealous people resort to sorcery to harm us. Therefore, we feel the need to perform reactive sorcery to counteract these negative effects.” (Woman, 46-years-old)

This suggests a deeper spiritual connection between Sri Lankan immigrants and their home country’s experiences, leading to the continued use of traditional beliefs. These beliefs play a vital role in ensuring a sense of normalcy and security in their new lives. Some immigrants utilize black magic rituals as a way to indirectly express anger and distress through a religious lens. For example:

“I faced a lot of trouble after migrating. My parents suggested that it was because some malicious individuals, driven by greed, had cursed me. This thought lingered in my mind for a long time. That’s why I consulted a Sri Lankan sorcerer to address these unfortunate occurrences.” (Man, 43-years-old)

The beliefs immigrants hold from their home country continue to impact their lives in the destination country. In some cases, negative narratives formed around these beliefs can destabilize their psychological well-being. To address these instabilities, immigrants may rely on mechanisms accepted in their home country, such as witchcraft and sorcery. These practices are believed to refine negative narratives and restore psychological stability, leading to subjective well-being. Specifically, black magic is often used to dispel and relieve internal turmoil associated with anger and hatred. This allows immigrants to reframe their perspectives and continue their immigration journey toward achieving their aspirations. Case studies in our study highlight the symbolic role of talismans and related totems in strengthening mental stability. For example, wearing chanted objects or wowed chains and bracelets is believed to offer protection from evil forces and bring success. While the factual basis of these beliefs may be debatable, their perceived truth plays a crucial role in determining emotional well-being. By positively refining these beliefs through religious engagement, individuals can achieve mental stability and subjective well-being.

The widespread use of white and black magic rituals among Sri Lankan immigrants in Italy show a unique cultural adaptation. It demonstrates how home country belief systems are appropriated and integrated into life in the destination country. The continuation of these religious practices holds significance for many immigrants. The instability encountered when navigating the host society can, in some cases, be addressed through the lens of familiar home country beliefs. These practices offer a sense of comfort and control in a potentially unfamiliar and challenging environment.

## 6 Discussion

Religious affiliations and spirituality can be vital ingredients of happiness, although cultural variations moderate their effects on subjective well-being. Studies by Lewis and Cruise (2006) and Frey (2018) found a positive correlation between religion and subjective well-being, suggesting that frequent church attendance and Protestant denominations have a particularly strong impact. Therefore, understanding the role of religion in determining subjective well-being is crucial, even for immigrants who bring their home country religious experiences to the new destination. While both our research and Sander (2017) acknowledge that the effect of religion on happiness is not universal, Vukcevic (2020) adds a new layer by suggesting that immigration can actually strengthen religious identity, potentially leading to greater happiness. However, existing research often overlooks the nature of religiosity and religious affiliation itself, focusing solely on religion and migration without considering the deeper cultural meanings attached to them.

Our research goes beyond this by proposing that the religious practices (habitus) brought from the home country can have a greater impact on immigrants’ lives than the religions prevalent in their new host country. In Bourdieu’s (2002) perspective, the ingrained practices are difficult to change, and we found that home country based religious practices of immigrants remerges even in the host society. It is important to note, however, that the evidence is mixed on the link between religiosity and happiness. While some studies show a positive association, others suggest that belonging to a formal religion can even decrease happiness (Hastings and Roeser, 2020). Therefore, sometimes the religious institutions act as barriers to freedom, which restrict individuals’ exploration of happiness (Laura, 2020). For immigrants, as our findings suggest, both religiosity and maintaining ties to a home country based religion are important factors in linking objective and subjective well-being.

Social cohesion is another well researched area at the intersection of migration and religion (Amit and Riss, 2014; Connor, 2012; Van Der Lans et al., 2000). Van Der Lans et al. (2000) demonstrates that religious adherence and subjective well-being are influenced by group cohesion, with religious practices strengthening a sense of community. Building upon this research, we argue that the cohesion fostered by religious affiliation can lead to the formation of distinct social milieus among immigrants, potentially hindering assimilation with the host population, instead promoting integration. As a result, relying solely on home country based religions might lead to a reluctance to engage with the host culture. However, our research highlights a counterpoint: home country based religious affiliations can contribute to psychological stability, thereby promoting subjective well-being.

Future studies should place greater emphasis on the longitudinal developments in religious affiliations and spirituality, even though this aspect was not a focus of our study. However, our findings suggest that age is a key determinant of religiosity among immigrants, with younger immigrants being less engaged in religious activities compared to older immigrants. Over time, various life course factors are likely to influence their affiliation with religious institutions. Some longitudinal studies support our findings, indicating that the significance of religion tends to increase the longer immigrants reside in the host country (Francis et al., 2003; Headey et al., 2010).

Studies on the migration-religion nexus have been overly optimistic and prone to overgeneralization, thereby obscuring the lived experiences of immigrants and the impact of cultural variations. For example, research suggests a counterintuitive finding: while spirituality can increase subjective well-being, religious affiliations might decrease happiness (Lewis and Cruise, 2006; Revens et al., 2021). However, as our findings highlights, this relationship is culturally contingent. Our participants demonstrated that both spirituality and religious affiliations can enhance resilience and subjective well-being. Contrary to previous studies suggesting a uniformity among immigrant groups (Connor, 2012; Formoso-Suárez et al., 2022; Simkin, 2020; Vishkin and Ben-Nun Bloom, 2022), our research reveals a unique case: Sri Lankan immigrants in Italy often exhibit characteristics of religious pluralism. However, a more precise term for their religious pluralism might be “ultimist-religious pluralism.” This is because they often identify with a primary religion while incorporating elements of other belief systems to fulfil their aspirations (McEntee, 2017). This finding suggests that the inherent uncertainty of immigrant lives, coupled with the desire to achieve aspirations by utilising the most advantageous resources, compels immigrants to adopt multiple religious belief systems, at least on a spiritual level.

While previous studies generally suggest that religion can increase happiness, our findings indicate a different mechanism for immigrants. Religion functions as a dynamic bridge between objective well-being (material conditions) and subjective well-being (feelings of happiness). Notably, home country based religions are particularly important in this regard. They provide a sense of cultural belonging, connecting immigrants to their community and past experiences. The bonding capital formed through religious gatherings helps expand existing networks, ultimately fostering resilience and happiness. A strong social network is a key determinant of subjective well-being. However, we did not find that religious institutions acting for ensuring bridging social capital, as they do not strive to connect immigrants into host societies. Rather, they work on connecting immigrants to home country based cultural experiences.

Our research also presents a unique perspective on the relationship between religion and the initial stages of immigration. Contrary to existing research on the religion-migration nexus (Formoso-Suárez et al., 2022; Koenig, 2012), we found that the need for religion to elevate subjective well-being is minimal during the first few years of immigration. Religious support is primarily sought when immigrants encounter uncertain or difficult life events. Udayanga (2020) theorized this pattern, suggesting that spiritual coping emerges as a way to positively reframe life experiences and promote psychological stability. Van Der Lans et al. (2000) observed a similar trend. However, we highlight that the need for religious affiliation and spirituality does emerge over time, after several years of migration. This is likely because, as some have suggested, the initial high levels of happiness experienced by immigrants tend to level off over time. Our analysis suggests that both spirituality and religious affiliations are crucial ingredients in linking immigrants’ objective well-being to their subjective well-being in the long term.

Unlike many studies that have examined the religion-migration nexus from the perspective of the destination country, our research contributes to the understanding of how home country based religion can be a source of happiness for immigrants. Our findings suggest that cultural disconnection (unlinking) does not immediately impact immigrants’ well-being. However, once they establish objective well-being over time, cultural disconnection can negatively affect their subjective well-being. At this point, immigrants often seek to reconnect with their home country based cultural experiences. This is because they may feel a sense of self discovery by aligning the objective well-being that they have achieved in the host country with their home country associated value system. Religious affiliations and spirituality based on their home country play a key role in facilitating this connection. While some have argued that historical religious connections between countries are important (Amit & Riss, 2014; Connor, 2012), our research suggests otherwise. We propose that regardless of historical connections, immigrants tend to draw on their religious experiences from their home country to navigate their new lives.

Several studies (Munas, 2023; Schmees et al., 2024; Vukcevic, 2020), along with our own research, highlight that migration can strengthen and crystallize an immigrants’ home country based religious identity. However, immigrants often attempt to adapt their religious traditions to fit the context of the host country. Despite maintaining a primary religious identity, we suggest that immigrants may be gravitating toward the ultimist religious pluralism. This concept, developed by Cobb and Griffin, posits that individuals can hold onto a core religion while also incorporating elements from other belief systems (McEntee, 2017). Our observations among immigrants suggest that their most valued religious beliefs may shift over time, depending on their needs. This flexibility represents a nuanced contribution to our study. Therefore, both real and anticipated uncertainties can motivate immigrants to seek support from religions with which they have prior experience.

Religious social capital is a well-researched concept, particularly among immigrant populations (Chen and Williams, 2016; Fry, 2023; Sanchez et al., 2019; Toma, 2016). Our research contributes to this discussion by demonstrating how home country based religions can provide immigrants with a stable foundation for developing strong social networks. These networks are essential for sharing experiences and integrating Sri Lankan cultural practices into the Italian social context. While not necessarily aligned with mainstream religious traditions, this trend involves incorporating local, traditional beliefs into the host environment—beliefs that are deeply woven into the lives of immigrants. Furthermore, we emphasize that religious affiliation alone does not guarantee happiness. However, the sense of belonging and the productive social networks fostered through religious affiliations create a supportive environment that promotes resilience and subjective well-being among immigrants. In contrast, spirituality that aligns with home country based beliefs emerges as a stronger predictor of subjective well-being for Sri Lankan immigrants in Italy.

There is a well-established connection between immigrant experiences and religious affiliations. Studies have shown that immigrants often report higher levels of happiness compared to natives, and this may be attributed to the role of spirituality and religious ties (Formoso-Suárez et al., 2022). Our research expands on this by demonstrating the importance of traditional belief systems, including witchcraft and sorcery, for some immigrants. These systems can sometimes be even more crucial for subjective well-being than their primary religious affiliation. The rituals associated with these traditional beliefs can significantly shape the aspirational pathways immigrants pursue in the host society. Although previous studies highlight the importance of immigrant acculturation for cultural and moral integration into the host society (Alegría et al., 2016; Bhugra et al., 2020), our findings indicate that immigrants, even after acculturation, often continue practising their home country based religions and beliefs. This persistence helps ensure that their perceived objective well-being aligns with their subjective sense of well-being. Affiliation with the religion and beliefs of the home country can support resilience and community belonging, yet simultaneously hinder effective integration of immigrants into host countries. However, this aspect was not addressed in our study and warrants further research.

Significant progress has been made in immigrant integration policies in Italy and, more broadly across Europe, in recent years. Nevertheless, cultural assimilation has received relatively little attention, despite its considerable potential to facilitate the integration of immigrants into host countries. When immigrants primarily engage with religious institutions associated with their home country rather than those of the host country. Therefore, future integration policies could benefit from recognising this relationship and incorporating immigrant-based religious organizations as key integration stakeholders, given their capacity to effectively address immigrant concerns.

We acknowledge several limitations in our study, as it specifically focused on voluntary Sri Lankan immigrants settled in Italy. As a result, our findings primarily represent those engaged in religious activities, whereas those without religious engagement may exhibit entirely different patterns. Nonetheless, our aim was to explore how active participation in religious activities linked to home country based values could promote social belonging and, consequently, subjective well-being. Although the nature of our research question limited our analysis to a particular immigrant community, the findings can be generalizable to communities with similar characteristics. Thus, while this study highlights the experiences of Sri Lankan immigrants in Italy, the insights gained can also inform our understanding of other immigrant groups sharing similar traits. We recommend further cross cultural studies and analyses of relationships within religious groups to better understand how diverse cultural backgrounds influence immigrant behaviors.

In conclusion, the spirituality and religious affiliation that immigrants retain from their home countries emerge as crucial determinants of their subjective well-being. While religious practices may not hold immediate significance during the initial stages of immigration, facing uncertainties can rekindle their importance. By fostering psychological stability, these practices ultimately contribute to both objective well-being and subjective well-being. Furthermore, achieving immigrant aspirations, while instrumental in attaining objective well-being, may not fully guarantee subjective well-being. It is through strong spiritual connections and religious affiliations that participants in our study can bridge this gap. Religion serves as a bridge, bringing familiar traditions and practices from the home country into the host society. This process of “relinking” with their heritage empowers immigrants to construct a fulfilling life, integrating objective well-being in the new environment while preserving their existing value systems. The conceptualization of subjective well-being is often established before migration and strongly influenced by values formed in the home country. Therefore, maintaining at least a virtual connection to these home country experiences is essential for life satisfaction, as immigrants’ current perception of well-being remains linked to concepts developed prior to migration.

## Data Availability

The dataset used for analysis is not publicly available except for the excerpts and de-identified photographs presented in the article, as participants did not provide consent for complete data sharing for the public use. They approved only the inclusion of essential narrative segments and anonymized images for publication. Therefore, any requests to access the qualitative dataset should be directed to the corresponding author and will be considered on a case-by-case basis, subject to ethical review and participant confidentiality.
